# SNPs related to vitamin D and breast cancer risk: a case-control study

**DOI:** 10.1186/s13058-017-0925-3

**Published:** 2018-01-02

**Authors:** Linnea Huss, Salma Tunå Butt, Peter Almgren, Signe Borgquist, Jasmine Brandt, Asta Försti, Olle Melander, Jonas Manjer

**Affiliations:** 1 Department of Surgery, Lund University, Skåne University Hospital, SE-205 02 Malmö, Sweden; 2 Department of Clinical Sciences, Malmö Clinical Research Centre, Lund University, Skåne University Hospital, SE-205 02 Malmö, Sweden; 3 Division of Oncology and Pathology, Department of Clinical Sciences, Lund University, Lund, Sweden; 4 Clinical Trial Unit, Skåne University Hospital, SE-221 85 Lund, Sweden; 5 Division of Molecular Genetic Epidemiology, German Cancer Research Centre (DKFZ), Im Neuenheimer Feld 580, D-69120 Heidelberg, Germany; 6 Centre for Primary Health Care Research, Lund University, SE-205 02 Malmö, Sweden

**Keywords:** Vitamin D, Breast cancer, Polymorphism, SNP

## Abstract

**Background:**

It has been suggested that vitamin D might protect from breast cancer, although studies on levels of vitamin D in association with breast cancer have been inconsistent. Genome-wide association studies (GWASs) have identified several single-nucleotide polymorphisms (SNPs) to be associated with vitamin D. The aim of this study was to investigate such vitamin D-SNP associations in relation to subsequent breast cancer risk. A first step included verification of these SNPs as determinants of vitamin D levels.

**Methods:**

The Malmö Diet and Cancer Study included 17,035 women in a prospective cohort. Genotyping was performed and was successful in 4058 nonrelated women from this cohort in which 865 were diagnosed with breast cancer. Levels of vitamin D (25-hydroxyvitamin D) were available for 700 of the breast cancer cases and 643 of unaffected control subjects. SNPs previously associated with vitamin D in GWASs were identified. Logistic regression analyses yielding ORs with 95% CIs were performed to investigate selected SNPs in relation to low levels of vitamin D (below median) as well as to the risk of breast cancer.

**Results:**

The majority of SNPs previously associated with levels of vitamin D showed a statistically significant association with circulating vitamin D levels. Heterozygotes of one SNP (*rs12239582*) were found to have a statistically significant association with a low risk of breast cancer (OR 0.82, 95% CI 0.68–0.99), and minor homozygotes of the same SNP were found to have a tendency towards a low risk of being in the group with low vitamin D levels (OR 0.72, 95% CI 0.52–1.00). Results from stratified analyses showed diverse associations with breast cancer risk for a few of the tested SNPs, depending on whether vitamin D level was high or low.

**Conclusions:**

SNPs associated with vitamin D may also be associated with the risk of breast cancer. Even if such a risk is small, the allele frequency of the SNP variants is high, and therefore the population attributable risk could be substantial. It is also possible that vitamin D levels may interact with genomic traits with regard to breast cancer risk.

**Electronic supplementary material:**

The online version of this article (doi:10.1186/s13058-017-0925-3) contains supplementary material, which is available to authorized users.

## Background

About 5–10% of breast cancers are considered hereditary, from which the known breast cancer genes account for 3–4% [1]. Apart from known breast cancer genes, genome-wide association studies (GWASs) have previously identified more than 170 genetic polymorphisms associated with the risk of breast cancer [2]. It has been suggested that single-nucleotide polymorphisms (SNPs) may add up to 14% of heredity of breast cancer [1].

Ecological and epidemiological studies have suggested a beneficial effect of relatively high vitamin D levels, owing to solar exposure, on breast cancer risk and survival [3–6]. There are several prospective epidemiological studies on the relationship between serum levels of vitamin D and breast cancer incidence. The results have been conflicting [7–10], although authors of a meta-analysis concluded that there is an inverse relationship between levels of vitamin D and breast cancer risk [11]. Diverse results may be a result of misclassification of vitamin D status, and a better marker of stable and unconfounded vitamin D status over time may be found in the genotype, as studied using a Mendelian randomization approach [12].

Researchers investigating vitamin D-related SNPs and breast cancer risk have focused mainly on SNPs located in the vitamin D receptor (VDR) gene. Results derived from these studies have also been conflicting [13–16]. SNPs of the VDR have not been found to be associated with vitamin D in previous GWASs [2]. The aim of the present study was to investigate breast cancer risk in relation to SNPs previously identified in GWASs on vitamin D levels and related phenotypes. The present study was a prospective, nested case-control study with information on SNPs, vitamin D levels and subsequent breast cancer.

## Methods

### Malmö diet and cancer study

Between 1991 and 1996, all residents of Malmö, Sweden, born between 1923 and 1950 were invited to participate in a prospective cohort study. Among invited women, 43% participated, resulting in a female cohort of 17,035 women [17]. At inclusion, written informed consent was obtained from all participants. Baseline examinations included anthropometric measurements by a trained nurse who also drew blood samples. Subjects were included evenly over the calendar year, although there was less recruitment in December and June and none in July. Participants also provided information on reproductive factors and lifestyle via a self-administered questionnaire. Information on previous gynaecological surgery was retrieved from medical records, and menopausal status was defined using these data together with information provided in the questionnaire [18]. The Malmö Diet and Cancer Study (MDCS) (LU 51-90) and the present study (Dnr 153/2004 and Dnr 682/2009) were approved by the regional ethics committee in southern Sweden.

### Study population

**Fig. 1:**
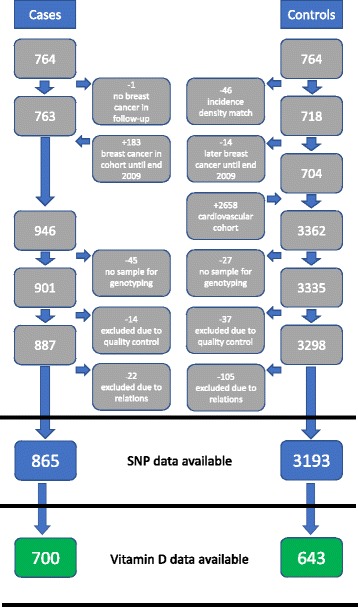
Flowchart of cases and control subjects. *SNP* Single-nucleotide polymorphism

In a previous case-control study, women diagnosed with breast cancer in the MDCS until December 31, 2006 (*n* = 764), were matched on age and date of inclusion with control subjects (*n* = 764), based on 1482 individuals (some were included twice because incidence density matching was applied) [8]. One breast cancer case was classified to be without disease in a later follow-up. Until December 31, 2009, an additional 183 women were diagnosed with breast cancer, leading to inclusion of 946 women with breast cancer in the MDCS cohort. The control group was expanded to include an additional 2658 randomly selected women from the MDCS cohort without a breast cancer diagnosis. These women were also part of the MDCS cardiovascular cohort, a randomized subsample of MDCS [19]. Together with the 704 control subjects from the previous case-control study who had not developed breast cancer, potential control subjects added up to 3362. Material was accessible for genotyping from 901 of the breast cancer cases and 3335 of the control subjects (Fig. 1).

Genotyping was performed using the HumanOmniExpressExome BeadChip and iScan System (Illumina, San Diego, CA, USA) during 2012–2013 at the Broad Institute of MIT and Harvard University (Cambridge, MA, USA) (*n* = 4058) and at the Clinical Research Centre, Skåne University Hospital, Malmö, Sweden (*n* = 178). Sample-based quality control (QC) included controls for call rate, > 95% of SNPs per individual, and control for excess heterozygosity, which led to exclusion of 51 individuals. Owing to first- and second-degree relationships between individuals, a further 127 individuals were excluded, and the relative with the highest call rate was kept in the study population. After QC and exclusion of relatives, 865 patients with breast cancer and 3193 control subjects were available for statistical analysis (Fig. 1). PLINK version 1.07 software was used for QC and exclusion of relatives.

### Levels of vitamin D

In the previous case-control study of the MDCS cohort mentioned above, researchers investigated, among other things, serum levels of vitamin D in relation to breast cancer risk [8]. For this study, high-pressure liquid chromatography was used to analyse 25-hydroxyvitamin D [25(OH)D] levels, and laboratory analysis was successful in 700 breast cancer cases and 643 control subjects in the present study population (Fig. 1). Amongst subjects with data on SNPs and vitamin D level (1343 in total), 320 women (165 cases, 155 control subjects) were also subjects in the cardiovascular cohort. There is no consensus regarding adequate levels of vitamin D, and the levels vary substantially over the year; thus it is not possible to clinically define a high or low vitamin D level on a single measurement [20]. In this study, the median level of vitamin D of each calendar month of sampling was used as a cut-off between low and high levels of vitamin D.

### Selecting SNPs

To find SNPs previously associated with vitamin D, a search in a GWAS catalogue using the search string “vitamin D” identified 20 SNPs [2, 21]. The researchers in these studies had evaluated associations between SNPs and levels of vitamin D (15 SNPs) [22–25], vitamin D insufficiency (9 SNPs) [26] and levels of vitamin D-binding protein (DBP) (3 SNPs) [27]. Nine of these SNPs were directly genotyped on the BeadChip used in our study. Using the SNAP web-based tool, we identified proxies on the basis of linkage disequilibrium (LD) and physical distance to selected SNPs for an additional eight SNPs [28]. When several proxies were found, the two with the highest LD and closest proximity to the selected SNP were used. Altogether, 20 SNPs from 10 different loci in the genome were tested for associations with vitamin D level and breast cancer risk: 2 from *ST6GALNAC3* in chromosome 1, 4 from group-specific component (*GC*) intron in chromosome 4, 1 from chromosome 6, 2 upstream of *NPY* in chromosome 7, 1 from proximity to *MGMT* in chromosome 10, 2 from unidentified genes, 2 from *PDE3B* intron, 2 from *CYP2R1*, 3 from *NADSYN1* in chromosome 11 and 1 from *MTMR4* in chromosome 17. A description of tested SNPs and their original GWAS proxy, as well as previous association with vitamin D, is provided in Additional file 1.

During QC, monomorphic SNPs due to lack of variation in a European population were excluded, as were SNPs deviating from Hardy-Weinberg equilibrium (*p* < 10^−6^) or if the variant call rate was < 95% in all samples. PLINK version 1.07 software was used for QC and identification of selected SNPs [29].

### Statistical methods

Cases and control subjects were compared in two different sets with regard to established and potential risk factors. The first set contained all cases and control subjects with available SNP data; the other set included only cases and control subjects in whom vitamin D had been analysed in the previous study [8]. Control groups and groups with and without SNPs and vitamin D data were also compared with regard to risk factors in order to investigate the risk of potential selection bias.

Unconditional binary logistic regression analyses were performed to investigate associations between SNPs and low levels of vitamin D (below median), as well as between selected SNPs and risk of breast cancer. ORs with 95% CIs were calculated using the homozygote for the major allele as the reference group. Stratification by low versus high levels of vitamin D was added to see if there were any differences in breast cancer risk between these groups based on genotypes. This analysis was tested for interaction of low versus high vitamin D level. All analyses were also adjusted for year of inclusion, for age at baseline and for established or potential risk factors for breast cancer, including level of education, type of occupation, age at menarche, age at first childbirth, exposure to oral contraceptives, exposure to hormone replacement therapy, height, body mass index (BMI), alcohol consumption and smoking. Missing values for adjustment factors were coded in a separate category and included in multivariable analyses.

## Results

### Potential confounders in cases and control subjects

Women with incident breast cancer were more often than control subjects to be non-manual labour workers (Table 1). This was applicable in both sets of study populations, although the difference between groups was smaller in the study population in which vitamin D levels were analysed. Another observation was that women with incident breast cancer in both study populations were more likely to be users of hormone replacement therapy and were also more likely to have been exposed to oral contraceptives (Table 1). In the complete study population, we also saw that women with incident breast cancer were more often included in the MDCS during the summer, had a higher level of education and were peri- or pre-menopausal to a greater extent at inclusion than women in the control group. These differences were not seen in the study population with analysed vitamin D levels, presumably owing to previous age and inclusion date matching of control subjects (Table 1).

**Table 1:** Baseline characteristics of breast cancer cases and control subjects

Factor	Category	Study population with SNP data (%)		Study population with data on vitamin D (%)	
		Control subjects (*n* = 3193)	Cases (*n* = 865)	Control subjects (*n* = 643)	Cases (*n* = 700)
Age, years	*Mean ± SD*	*57.4 ± 6.3*	*56.6 ± 7.3*	*57.0 ± 7.3*	*56.9 ± 7.2*
Season of inclusion in MDCS	January–March	33.1	28.8	29.9	29.9
	April–June	19.0	24.7	24.0	23.9
	July–September	18.0	22.8	21.3	21.9
	October–December	29.9	23.7	24.9	24.4
Education	O-level college (7–9 years)	73.6	66.5	68.0	67.7
	A-level college (11–12 years)	6.8	7.1	7.3	7.3
	University	19.4	26.1	24.7	24.7
Type of occupation	Manual labour worker	39.6	32.7	38.7	34.4
	Non-manual labour worker	52.5	60.6	53.8	58.6
	Employer/self-employed	6.9	5.7	7.5	5.7
	Missing	0.9	1.0	0.0	1.3
Married/co-habitating	No	31.0	33.5	32.0	34.0
	Yes	69.0	66.5	68.0	66.0
Age at menarche, years	< 12	6.2	7.6	4.5	7.3
	12–15	67.2	67.3	68.9	67.1
	> 15	25.6	23.9	26.0	24.4
	Missing	0.9	1.2	0.6	1.1
Parity	Nulliparous	12.0	14.1	11.2	13.6
	One child	21.4	19.5	22.4	19.3
	Two children	40.6	44.7	40.9	44.1
	Three children or more	22.8	19.5	22.7	20.6
	Missing	3.1	2.1	2.8	2.4
Age at first birth, years	Nulliparous	12.0	14.1	11.2	13.6
	≤ 20	16.5	14.9	17.9	13.6
	21–24	28.2	27.3	27.7	27.7
	25–29	29.1	28.7	28.6	30.1
	≥ 30	11.1	12.9	11.8	12.6
	Missing	3.1	2.1	2.8	2.4
Age at menopause, years	Pre-/peri-menopause	27.2	35.6	32.5	33.1
	< 45	9.8	9.0	8.6	9.4
	45–53	45.3	39.5	42.6	41.6
	> 53	16.0	13.8	14.9	13.7
	Missing	1.7	2.1	1.4	2.1
Exposure to oral contraceptives	No	54.3	45.9	50.1	48.6
	Yes	45.7	54.0	49.9	51.3
Exposure to hormone replacement therapy (HRT)	No (pre-menopausal)	22.0	28.4	26.3	25.7
	No (post-menopausal)	59.8	44.5	53.3	45.7
	Oestrogen only	7.6	7.4	9.2	7.6
	Progesterone only	0.6	1.2	0.9	1.3
	Combined HRT	9.4	18.3	9.8	19.4
Height, m	≤ 1.59	24.6	20.6	23.2	20.3
	1.60–1.69	58.1	61.4	59.1	61.6
	≥ 1.70	17.2	18.0	17.7	18.1
Body mass index, kg/m^2^	< 25	53.3	50.1	51.8	50.6
	≥ 25 to < 30	33.6	34.5	36.1	33.3
	≥ 30	13.0	15.5	12.1	16.1
Alcohol consumption	Not in last year	11.4	9.7	11.4	10.4
	Some in last year	12.7	11.2	12.4	11.4
	Some in last month	75.6	78.8	75.9	78.0
Smoking	Never	46.8	43.0	42.5	44.0
	Current	26.7	26.7	27.8	26.6
	Ex-smoker	26.4	30.3	29.7	29.4

*MDCS* Malmö Diet and Cancer Study, *SNP* Single-nucleotide polymorphism

Separate missing categories are given only if missing > 1%, Mean ± SD in italics

When comparing control subjects from the cardiovascular cohort with control subjects used in the previous case-control study, risk factors were similarly distributed, although small differences in a higher level of education and a slightly higher proportion of oral contraceptive users and smokers were seen amongst the previously matched control subjects (Additional file 2). Women whose data were excluded owing to QC of SNPs or relationships with other women in the cohort were less likely to have used oral contraceptives. They also had a lower BMI and had not used tobacco to the same extent as women with included data (Additional file 2). There also were small differences between women with or without available data on vitamin D level. Women with no data on vitamin D used oral contraceptives and hormone replacement therapy less and were also slightly older and less likely to smoke (Additional file 2).

### SNPs and risk of low vitamin D level

There were statistically significant associations between a majority of tested SNPs and levels of vitamin D (Table 2). An increased risk of being in the group with lower vitamin D was seen for minor homozygotes of *rs705117* (OR 3.38, 95% CI 1.20–9.49), rs7041 (OR 2.12, 95% CI 1.51–2.99) and *rs2282679* (OR 2.51, 95% CI 1.56–4.05), all of which are located in the intron of the GC gene on chromosome 4 (Table 2). For *rs7041* and* rs2282679*, the association was seen also for heterozygotes (respectively, OR 1.39, 95% CI 1.09–1.77; OR 1.42, 95% CI 1.13–1.80) (Table 2). Similarly, higher risk of low vitamin D was associated with minor homozygotes of *rs12295888* (OR 1.80, 95% CI 1.24–2.63), *rs1007392* (OR 1.78, 95% CI 1.26–2.52), rs7944926 (OR 2.39, 95% CI 1.59–3.59) and rs3829251 (OR 3.26, 95% CI 1.72–6.17) on chromosome 11 (Table 2). A statistically significant decreased risk of low vitamin D was seen with heterozygotes (OR 0.78, 95% CI 0.61–1.00) and minor homozygotes (OR 0.58, 95% CI 0.42–0.81) of rs2060793 also located on chromosome 11 but in the *CYP2R1* gene (Table 2).

**Table 2:** Selected single-nucleotide polymorphisms in relation to vitamin D level and breast cancer

SNP (GWAS SNP)	Risk of low vitamin D			Risk of breast cancer		
Allele	Frequency of low/high vitamin D^a^ (*n* = 1343)	Crude OR of low vitamin D (95% CI)	Adjusted OR^b^ of low vitamin D (95% CI)	Cases/control subjects (*n* = 4058)	Crude OR (95% CI)	Adjusted OR^b^ (95% CI)
rs12239582 (rs12144344)						
CC	224/188	1.00	1.00	290/986	1.00	1.00
CA	344/336	0.86 (0.67–1.10)	0.87 (0.67–1.12)	412/1604	0.87 (0.74–1.04)	0.82 (0.68–0.99)
AA	116/135	0.72 (0.53–0.99)	0.72 (0.52–1.00)	163/600	0.92 (0.74–1.15)	0.93 (0.73–1.17)
Missing	–	–	–	0/3	–	–
rs705117						
CC	504/486	1.00	1.00	657/2376	1.00	1.00
CT	164/168	0.94 (0.73–1.21)	0.94 (0.73–1.22)	194/762	0.92 (0.77–1.10)	0.86 (0.71–1.05)
TT	16/5	3.06 (1.11–8.44)	3.38 (1.20–9.49)	14/55	0.92 (0.51–1.67)	0.94 (0.49–1.81)
rs7041						
AA	213/270	1.00	1.00	332/1149	1.00	1.00
AC	341/308	1.40 (1.11–1.78)	1.39 (1.09–1.77)	397/1521	0.90 (0.77–1.07)	0.88 (0.74–1.05)
CC	130/81	2.03 (1.46–2.83)	2.12 (1.51–2.99)	136/522	0.90 (0.72–1.13)	0.89 (0.70–1.13)
Missing	–	–	–	0/1	–	–
rs2282679 (rs17467825)						
GG	342/397	1.00	1.00	480/1734	1.00	1.00
GT	278/230	1.40 (1.12–1.76)	1.42 (1.13–1.80)	326/1222	0.96 (0.82–1.13)	0.98 (0.83–1.16)
TT	62/29	2.45 (1.56–3.95)	2.51 (1.56–4.05)	57/229	0.90 (0.66–1.22)	0.91 (0.66–1.27)
Missing	2/3	–	–	2/8	–	–
rs10485165						
TT	515/501	1.00	1.00	650/2385	1.00	1.00
TC	153/144	1.04 (0.80–1.35)	1.03 (0.79–1.34)	194/735	0.97 (0.81–1.16)	0.99 (0.82–1.20)
CC	16/14	1.10 (0.53–2.29)	1.04 (0.49–2.21)	21/73	1.06 (0.65–1.73)	1.00 (0.60–1.74)
rs198300 (rs156299)						
GG	218/229	1.00	1.00	295/1134	1.00	1.00
GA	344/319	1.13 (0.89–1.44)	1.08 (0.84–1.38)	430/1513	1.09 (0.92–1.29)	1.10 (0.91–1.30)
AA	122/111	1.16 (0.84–1.59)	1.09 (0.79–1.52)	140/545	0.99 (0.79–1.24)	0.96 (0.75–1.22)
Missing	–	–	–	0/1	–	–
rs4751058						
AA	496/460	1.00	1.00	614/2286	1.00	1.00
AG	174/183	0.88 (0.69–1.13)	0.88 (0.68–1.13)	234/828	1.05 (0.89–1.25)	1.05 (0.88–1.27)
GG	14/16	0.81 (0.39–1.68)	0.77 (0.36–1.63)	17/79	0.80 (0.47–1.36)	0.66 (0.38–1.17)
rs12295888 (rs12287212)						
CC	270/296	1.00	1.00	367/1384	1.00	1.00
CT	321/302	1.16 (0.93–1.46)	1.22 (0.96–1.54)	402/1435	1.06 (0.90–1.24)	1.05 (0.88–1.24)
TT	93/61	1.67 (1.16–2.40)	1.80 (1.24–2.63)	96/373	0.97 (0.75–1.25)	0.93 (0.71–1.23)
Missing	–	–	–	0/1	–	–
rs1007392						
GG	231/267	1.00	1.00	325/1184	1.00	1.00
GA	337/310	1.26 (0.99–1.59)	1.31 (1.03–1.67)	417/1543	0.99 (0.84–1.16)	0.99 (0.83–1.18)
AA	115/82	1.62 (1.16–2.26)	1.78 (1.26–2.52)	123/462	0.97 (0.77–1.23)	0.91 (0.71–1.17)
Missing	1/0	–	–	0/4	–	–
rs2060793 (rs10741657)						
AA	246/194	1.00	1.00	283/1040	1.00	1.00
AG	328/323	0.80 (0.63–1.02)	0.78 (0.61–1.00)	418/1544	1.00 (0.84–1.18)	1.10 (0.92–1.33)
GG	110/142	0.61 (0.45–0.84)	0.58 (0.42–0.81)	164/606	1.00 (0.80–1.24)	1.04 (0.82–1.32)
Missing	–	–	–	0/3	–	–
rs7944926 (rs12785878)						
AA	297/343	1.00	1.00	423/1436	1.00	1.00
AG	299/272	1.27 (1.01–1.59)	1.30 (1.03–1.64)	366/1400	0.89 (0.76–1.04)	0.91 (0.77–1.08)
GG	88/44	2.31 (1.56–3.43)	2.39 (1.59–3.59)	76/356	0.73 (0.55–0.95)	0.77 (0.57–1.02)
Missing	–	–	–	0/1	–	–
rs3829251						
AA	402/435	1.00	1.00	545/1954	1.00	1.00
AG	242/210	1.25 (0.99–1.57)	1.30 (1.02–1.64)	287/1079	0.95 (0.81–1.12)	0.99 (0.83–1.18)
GG	40/14	3.09 (1.65–5.77)	3.26 (1.72–6.17)	33/160	0.74(0.50–1.09)	0.77 (0.51–1.16)
rs2302190						
CC	451/466	1.00	1.00	566/2122	1.00	1.00
CT	211/176	1.24 (0.98–1.57)	1.24 (0.97–1.58)	268/955	1.05(0.89–1.24)	1.09 (0.91–1.30)
TT	20/13	1.59 (0.78–3.23)	1.45 (0.69–3.02)	28/109	0.96 (0.63–1.47)	0.99 (0.62–1.58)
Missing	2/4	–	–	3/7	–	–

*GWAS* Genome-wide association study, *SNP* Single-nucleotide polymorphism

^a^Low vs high vitamin D grouped by calendar month

^b^Adjusted for age at baseline, year of inclusion, level of education, type of occupation, age at menarche, age at first childbirth, exposure to oral contraceptives, exposure to hormone replacement therapy, height, body mass index, alcohol consumption and smoking

When several proxies were used for one GWAS SNP, the results in the analyses were very similar, but only the proxy with an *R* ^2^ value closest to 1 is presented in this report.

### SNPs and overall breast cancer risk

The tested SNP proxy *rs12239582* for the GWAS SNP *rs12144344* (located in *ST6GALNAC3* in chromosome 1) was found to be statistically significantly associated with a relatively low risk of breast cancer (OR 0.82, 95% CI 0.68–0.99) in the adjusted model when heterozygotes were compared with major homozygotes (Table 2). In the crude analysis, minor homozygotes of SNP proxy *rs12791871* for GWAS SNP *rs12785878* (located in *NADSYN1* in chromosome 11) also had a statistically significant decreased risk of breast cancer (OR 0.73, 95% CI 0.55–0.95) compared with major homozygotes (Table 2). This result was similar in the adjusted analysis, although with only borderline statistical significance (OR 0.77, 95% CI 0.57–1.02) (Table 2).

### SNPs and breast cancer risk in different strata of serum vitamin D

A test of interaction of high/low vitamin D levels and breast cancer risk was statistically significant at *p* < 0.01 for *rs198300* (proxy for GWAS *rs156299*, located upstream of *neuropeptide Y [NPY]* gene in chromosome 7). Minor homozygotes of *rs198300* who were within the group with low vitamin D levels had a statistically significant decrease in breast cancer risk compared with major homozygotes (OR 0.53, 95% CI 0.33–0.87) (Table 3). Looking at minor homozygotes of the same SNP (rs198300) within the group with high vitamin D levels, their risk of breast cancer seemed to be increased (OR 1.38, 95% CI 0.85–2.23) (Table 3). Similar but not statistically significant results were seen for *rs2060793* (proxy for GWAS *rs10741657* located in gene encoding cytochrome P450 *[CYP2R1]* in chromosome 11); compared with major homozygotes, heterozygotes in the group with lower levels of vitamin D had a slightly decreased risk of breast cancer (OR 0.95, 95% CI 0.67–1.35), but in the high vitamin D group, a reverse effect with increased breast cancer risk was seen (OR 1.35, 95% CI 0.93–1.97; *p* = 0.10 for interaction) (Table 3).

**Table 3:** Selected single-nucleotide polymorphisms in relation to risk of breast cancer, stratified by vitamin D level

SNP (proxy for GWAS)	Low vitamin D^a^			High vitamin D^a^	
Allele	Frequency in cases/control subjects (*n* = 684)	OR^b^ of breast cancer (95% CI)	*p* Value for interaction	Frequency in cases/control subjects (*n* = 659)	OR^b^ of breast cancer (95% CI)
rs12239582 (rs12144344)					
CC	127/97	1.00		99/89	1.00
CA	176/168	0.85 (0.59–1.21)	0.73	164/172	0.91 (0.62–1.33)
AA	60/56	0.83 (0.52–1.34)	0.30	74/61	1.16 (0.72–1.87)
rs705117					
CC	278/226	1.00		251/235	1.00
CT	76/88	0.65 (0.45–0.95)	0.28	84/84	0.87 (0.60–1.27)
TT	9/7	1.14 (0.41–3.22)	0.36	2/3	0.45 (0.66–3.11)
rs7041					
AA	120/93	1.00		141/129	1.00
AC	169/172	0.70 (0.48–1.00)	0.14	161/147	1.05 (0.74–1.47)
CC	74/56	1.11 (0.69–1.76)	0.15	35/46	0.65 (0.38–1.11)
rs2282679 (rs17467825)					
GG	174/168	1.00		209/188	1.00
GT	155/123	1.25 (0.89–1.75)	0.16	113/117	0.91 (0.65–1.29)
TT	33/29	1.21 (0.68–2.16)	0.69	14/15	0.92 (0.42–2.04)
Missing	1/1	–	–	1/2	–
rs10485165					
TT	267/248	1.00		262/239	1.00
TC	88/65	1.34 (0.91–1.98)	0.08	67/77	0.83 (0.56–1.22)
CC	8/8	0.94 (0.32–2.78)	0.79	8/6	1.11 (0.36–3.40)
rs198300 (rs156299)					
GG	126/92	1.00		110/119	1.00
GA	184/160	0.82 (0.57–1.18)	0.25	165/154	1.06 (0.74–1.52)
AA	53/69	0.53 (0.33–0.87)	<0.01	62/49	1.38 (0.85–2.23)
rs4751058					
AA	267/229	1.00		235/225	1.00
AG	89/85	0.91 (0.63–1.329	0.37	96/87	1.06 (0.74–1.52)
GG	7/7	1.00 (0.33–3.03)	0.42	6/10	0.47 (0.16–1.38)
rs12295888 (rs12287212)					
CC	146/124	1.00		157/139	1.00
CT	169/152	0.94 (0.66–1.33)	0.88	154/148	0.86 (0.61–1.20)
TT	48/45	1.01 (0.60–1.67)	0.33	26/35	0.65 (0.36–1.17)
rs1007392					
GG	129/102	1.00		140/127	1.00
GA	177/160	0.87 (0.61–1.25)	0.71	159/151	0.91 (0.64–1.29)
AA	57/58	0.81 (0.50–1.30)	0.98	38/44	0.79 (0.47-1.34)
Missing	0/1	–	–		–
rs2060793 (rs10741657)					
AA	134/112	1.00		89/105	1.00
AG	168/160	0.95 (0.67–1.35)	0.10	174/149	1.35 (0.93–1.97)
GG	61/49	0.99 (0.61–1.60)	0.44	74/68	1.31 (0.83–2.08)
rs7944926 (rs12785878)					
AA	167/130	1.00		180/163	1.00
AG	158/141	0.88 (0.62–1.24)	0.75	137/135	0.93 (0.66–1.29)
GG	38/50	0.58 (0.35–0.98)	0.49	20/24	0.78 (0.40–1.51)
rs3829251					
AA	218/184	1.00		222/213	1.00
AG	126/116	0.95 (0.68–1.34)	0.56	110/100	1.07 (0.76–1.51)
GG	19/21	0.74 (0.37–1.49)	0.62	5/9	0.57 (0.18–1.79)
rs2302190					
CC	236/215	1.00		229/237	1.00
CT	114/97	1.03 (0.73–1.47)	0.49	97/79	1.27 (0.88–1.84)
TT	12/8	1.36 (0.51–3.60)	0.52	9/4	2.11 (0.61–7.30)
Missing	1/1	–	–	2/2	–

*GWAS* Genome-wide association study, *SNP* Single-nucleotide polymorphism

^a^Low vs high vitamin D grouped by calendar month

^b^Adjusted for age at baseline, year of inclusion, level of education, type of occupation, age at menarche, age at first childbirth, exposure to oral contraceptives, exposure to hormone replacement therapy, height, body mass index, alcohol consumption and smoking

Adverse associations of breast cancer risk dependent on vitamin D levels were seen for heterozygotes of *rs10485165* (in chromosome 6). Heterozygotes with low levels of vitamin D were associated with an increased risk of breast cancer (OR 1.34, 95% CI 0.91–1.98), whereas heterozygotes with higher vitamin D levels had a decreased risk of breast cancer (OR 0.83, 95% CI 0.56–1.22), with a *p* value for interaction of 0.08 (Table 3).

## Discussion

In the present study, a majority of the selected SNPs were associated with vitamin D levels that were in agreement with those reported in previous studies. As for breast cancer risk, a statistically significant association was observed with one of the tested loci (*rs12239582* and *rs2209458*; proxies for GWAS SNP *rs12144344*). Another locus (*rs12791871* and *rs7944926*; proxies for GWAS SNP *rs12785878*) showed a tendency towards an association with breast cancer risk. The analyses stratified for level of vitamin D showed diverse associations with breast cancer risk for three of the tested loci: *rs156299* (proxies used, *rs198300* and *rs13245518*), *rs10741657 *(proxies used, *rs2060793* and *rs1993116*) and *rs10485165*, depending on whether vitamin D level was high or low.

### SNPs and risk of low vitamin D

There is no current consensus regarding which level of vitamin D should be considered insufficient; in addition, the level varies over the year owing to sun exposure. Grouping individuals by high or low vitamin D level by calendar month was considered pragmatic to calculate differences in vitamin D level owing to allele variants. SNPs located in the intron of the *GC* gene in chromosome 4 have previously been associated in several studies with levels of vitamin D and/or vitamin D insufficiency [22, 23, 25–27]. Results derived from the present analyses are consistent with previous results because minor alleles of all tested SNPs located in the intron of the *GC* gene (*rs705117, rs7041, rs4588* and *rs2282679*) were statistically significantly associated with a low level of vitamin D. Equally confirming results were seen for several other SNPs of other genome sites, such as *PDE3B* intron, *CYP2R1*, and *NADSYN1* in chromosome 11. Borderline statistically significant associations were seen for further SNPs on *ST6GALNAC3* in chromosome 1 and on *MTMR4* in chromosome 17. SNPs in chromosome 6, chromosome 7 and chromosome 10 did not show any association with low vitamin D, in contrast to previous studies of children in Western Australia [23, 24]. This might depend on the different genetic backgrounds of the two populations, limited statistical power due to small groups with vitamin D levels in our material, or different vitamin D exposures because it is expected that children in Western Australia are likely to have different exposure to sun than middle-aged women in the south of Sweden.

If there is a true association between low levels of vitamin D and an increased risk of breast cancer, a Mendelian randomization study would show that SNPs associated with low vitamin D levels would also be associated with an increased risk of breast cancer [12]. This is somewhat in line with the present results derived from analysing *rs12239582* and *rs2209458* in *ST6GALNAC3*, in chromosome 1, where minor homozygotes had a borderline statistically significant association with a decreased risk of low vitamin D level and heterozygotes with a statistically significant decreased risk of breast cancer (Table 2). Contradictory to this, SNPs whose minor alleles showed an association with an increased risk of low vitamin D—*rs12791871, rs7944926* and *rs3829251*—of the *NADSYN1* in chromosome 11 were, if anything, associated with a decreased risk of breast cancer. Also, for most SNPs with an association with vitamin D level, no association with breast cancer risk could be found. This might be explained either by no such association being able to be found or by such an association being too small to be observed in our study. The clinical relevance of such small associations is questionable.

### SNPs and risk of breast cancer

The association found in our study between *rs12144344* (proxies, *rs12239582* and *rs2209458*) in chromosome 1 and breast cancer risk has not been reported previously. This SNP is positioned in *ST6GALNAC3*, a gene encoding a sialyltransferase which might affect DBP synthesis, concentration and function and that has previously been associated with levels of DBP [27]. No statistically significant association with vitamin D level was seen in our study, although minor homozygotes had a borderline statistically significant association with low levels of vitamin D. Previous associations between *rs12144344* and vitamin D levels have not been reported.

Previous studies on vitamin D-associated SNPs and breast cancer risk have been focused mainly on genetic variants of the vitamin D receptor, and a few studies have been done on *GC*, which is the gene encoding DBP [30]. The present study was focused on SNPs previously associated with vitamin D in GWASs, and because no association was previously found for VDR SNPs, no VDR SNPs were included. Of the SNPs analysed in this study, only *rs7041* and *rs4588*, both located in the GC gene, were previously studied in association with breast cancer risk [31–35]. Our results showing a tendency towards a protective effect of one or two minor alleles of *rs7041* and no association of *rs4588* are consistent with those of one previous study in which researchers showed a similar statistically significant association [32], as well as one other study in which researchers combined variants of *rs7041* and *rs4588* and found a protective effect of some combinations for post-menopausal breast cancer [36]. However, other studies have shown no association [30, 33, 35].

In the present study, the common homozygote was used as a reference, and relative risks were calculated individually for heterozygotes and less common homozygotes. Others have used other approaches, calculating on the basis of risk alleles and/or grouping several genotypes together. Sometimes it is not clear which allele was used as a reference, which means that comparing results between studies is difficult.

### SNPs and breast cancer risk in different strata of serum vitamin D

When the association of analysed SNPs with breast cancer risk was stratified by level of vitamin D, some results differed depending on vitamin D level. This association was found for *rs198300* and *rs13245518*, both proxies for *rs156299* located on chromosome 7 upstream of gene encoding neuropeptide Y, a neurotransmitter involved in mediating physiological processes, including food intake and bone homeostasis, previously associated with levels of vitamin D [23]. In non-stratified analyses, no association with either breast cancer risk or vitamin D level was observed. Similar results were found for *rs2060793* and *rs1993116*, proxies for GWAS* rs10741657* located in *CYP2R1*, which encodes cytochrome P450, an enzyme that converts vitamin D to the circulating form 25(OH)D. *rs10741657* was previously associated with vitamin D levels and insufficiency [22, 23, 25, 26]. Also, *rs10485165*, a proxy for *rs7763511* (in chromosome 6) previously associated with vitamin D level but with unknown function [24], showed diverse ORs for breast cancer risk dependent on high versus low vitamin D level.

We found no previous reports of associations of this kind. Because this was an exploratory part of the present study, and because vitamin D level was available for only a smaller fraction of cases and control subjects, we suggest that these findings ought to be regarded with caution. The findings must be replicated, but at the same time they indicate that vitamin D level may interact with genetic traits in breast cancer risk.

### Methodological issues

All patients diagnosed with cancer within the boundaries of Sweden are reported to the Swedish Cancer Registry, which is regarded as a highly validated source of information [37]. Thanks to the unique civil registration number given to all Swedes at birth, it is possible to link individuals included in the cohort to this registry and retrieve complete and correct information on breast cancer diagnosis. Women who have emigrated and received their diagnosis elsewhere are not included in this registry, which is a limitation of the present study; hence a few cancer cases might be lost, but emigration is not likely to be linked to risk of breast cancer or genetic traits.

An individual’s vitamin D level is influenced by several factors, not only sun exposure. It has also been shown that levels decrease with increasing age and increasing BMI [38–40], which was taken into consideration when analyses were adjusted. Physical activity has been shown to influence both level of vitamin D and breast cancer incidence [41, 42], but because the validity of physical activity data in the MDCS has been questioned, this was not included in the statistical model. Moreover, the effect of physical activity on vitamin D levels seems to be limited to outdoor activity, and hence physical activity may be a marker of sun exposure [43]. Vitamin D levels were analysed in only one blood sample per individual, which might be considered insufficient, though it has been shown previously that 25(OH)D levels analysed twice several years apart are individually very highly correlated [44, 45].

Differences noted when study populations were compared with regard to risk (Additional file 2: Table S1) were small. Those differences might depend on the fact that the previously matched control subjects had more in common with breast cancer cases. Considering differences between subjects with or without data regarding vitamin D level, there are of course a larger proportion of breast cancer cases amongst women with available vitamin D levels, which explains some of the differences. Also, because we adjusted analyses for risk factors in which subjects differ, we consider the risk of potential selection affecting our results to be small.

It is not plausible that any confounder included in this study affect an individual’s genetic composition. It might therefore be debatable whether adjustments should be added to the analyses. Because there is still a possibility of a chance association between single SNPs and established and potential risk factors for breast cancer, we chose to include such factors in our statistical model. A limitation of the present study is that there were no data accessible regarding family history of breast cancer or prior benign breast disease; hence those known risk factors were not included when adjustments were made.

In total, 20 SNPs from 10 different loci were tested for associations with breast cancer risk and risk of low vitamin D level in the present study. For each one of these, we used the same assumption: If there is an association with vitamin D, there might also be one with risk of breast cancer. We therefore decided not to increase the CI. With so many tested SNPs, one might suspect associations to be chance findings as a type I error, but when such associations were found, we also saw the same association for proxy SNPs, which strengthens the findings.

Individual SNPs change the risk of getting breast cancer to a very small extent, and large groups are a must in order to find statistically significant associations. Even the present population with 865 cases and 3193 control subjects may be too small to identify all associations. In the population with analysed levels of vitamin D, groups were even smaller, and therefore we expect a higher risk of a type II error in these analyses.

## Conclusions

Many previous findings of SNPs associated with vitamin D levels were reassuringly replicated in this study. One SNP (*rs12239582*) previously associated with levels of DBP was associated with a slightly decreased risk of breast cancer, as was a tendency towards a decreased risk of low vitamin D level, in the present study. The allele frequency of the SNP variants is high, and therefore even a small increase in risk per individual may be a substantial population attributable risk. Further results of the present study show vitamin D level to be an effect modifier of the risk of breast cancer associated with certain SNPs. This suggests that an individual’s composition of SNPs may affect the extent to which levels of vitamin D are associated with breast cancer risk.

## Additional files

Additional file 1: Appendix. Description of GWAS SNPs associated with vitamin D, SNP proxies and SNPs analysed. (DOCX 27 kb)

Additional file 2: Table S1. Baseline characteristics of control subjects and subjects with and without data on SNPs/vitamin D. (DOCX 24 kb)

## Abbreviations

BMI: Body mass index
DBP: Vitamin D-binding protein
GC: Group-specific component
GWAS: Genome-wide association study
HRT: Hormone replacement therapy
LD: Linkage disequilibrium
MDCS: Malmö Diet and Cancer Study
QC: Quality control
SNP: Single-nucleotide polymorphism
VDR: Vitamin D receptor
