# Supplementary material for: SNPs related to vitamin D and breast cancer risk: a case-control study
Source: Breast Cancer Res. 2018 Jan 2;20:1. doi: 10.1186/s13058-017-0925-3 (PMC5748964; doi:10.1186/s13058-017-0925-3)
Supplement: Supplementary file 1 — Appendix. Description of GWAS SNPs associated with vitamin D, SNP proxies and SNPs analysed. (DOCX 27 kb) [file 13058_2017_925_MOESM1_ESM.docx]

Additional file 1

| **Appendix:** Description of GWAS SNPs associated with vitamin D, SNP proxies and SNPs analyzed. | | | | | |
| --- | --- | --- | --- | --- | --- |
| SNP associated with vitamin D in GWAS | Association found | Reported gene | Proxy SNPs found in SNAP search | SNP available for analyses | Chromosome: Coordinate  According to Humon Genome Annotation 18 |
| **rs12144344** | Associated with levels of DBP^[[1]](#footnote-1)^[1] | ST6GALNAC3^[[2]](#footnote-2)^ | rs12239582  rs2209458 | rs12239582  rs2209458 | chr1:76609825 chr1:76635236 |
| **rs705117** | Associated with levels of DBP[1] | GC^[[3]](#footnote-3)^ intron | On chip | rs705117 | chr4:72826979 |
| **rs7041** | Associated with levels of DBP[1]  Associated with levels of vitamin D[2, 3] Associated with vitamin D insufficiency[4] | GC intron | On chip | **rs7041** | chr4:72837198 |
| **rs1155563** | Associated with levels of vitamin D[2, 3, 5] Associated with vitamin D insufficiency[4] | GC intron | rs4588 | rs4588 | chr4:72837187 |
| **rs2282679** | Associated with levels of vitamin D[2, 3, 5] Associated with vitamin D insufficiency[4] | GC intron | On chip | **rs2282679** | chr4:72827247 |
| **rs17467825** | Associated with levels of vitamin D[3, 5] Associated with vitamin D insufficiency[4] | GC intron | rs4588  rs2282679 | rs4588  rs2282679 | chr4:72837187 chr4:72827247 |
| **rs10485165** | Associated with vitamin D level[6] | Not identified | On chip | rs10485165 | chr6:89169536 |

| **rs156299** | Associated with levels of vitamin D determined by EIA^[[4]](#footnote-4)^, suggestive on standardized levels[5] | Upstream of NPY^[[5]](#footnote-5)^ | rs198300 rs13245518 rs156310  rs156282  rs19830 | rs198300  rs13245518 | chr7:24162708 chr7:24210537 |
| --- | --- | --- | --- | --- | --- |
| **rs4751058** | Associated with vitamin D level (not found in article text)[5] | (MKLN1) MGMT^[[6]](#footnote-6)^ | On chip | rs4751058 | chr10:130764115 |
| **rs12287212** | Associated with vitamin D level (not found in article text)[5] | Not identified | rs12295888 rs10832275 rs11023246 | rs12295888  rs10832275 | chr11:14407107  chr11:14434800 |
| **rs1007392** | Associated with levels of vitamin D[5] Proxy associated with levels of vitamin D[3] Proxy associated with vitamin D insufficiency[4] | PDE3B intron^[[7]](#footnote-7)^ | On chip | rs1007392 | chr11:14731167 |
| **rs11023332** | Associated with levels of vitamin D[5] Proxy associated with levels of vitamin D[3] Proxy associated with vitamin D insufficiency[4] | PDE3B intron | rs1007392 rs10832299 rs10832294 rs7938266 rs4757261 rs4757269 rs11023350 rs10500804 rs12794714 | rs1007392  rs10832299 | chr11:14731167 chr11:14725968 |
| **rs10741657** | Associated with levels of vitamin D[2, 3] Suggested association with vitamin D levels[5] Associated with vitamin D insufficiency[4]  Associated with level of vitamin D but not breast cancer risk[3] | CYP2R1^[[8]](#footnote-8)^ | rs2060793 rs1993116 rs1868997 rs7116978 rs6486205 | rs2060793  rs1993116 | chr11:14871886  chr11:14866810 |
| **rs2060793** | Associated with levels of vitamin D[2, 3] Associated with vitamin D insufficiency[4] | CYP2R1 | On chip | rs2060793 | chr11:14871886 |
| **rs12785878** | Associated with vitamin D insufficiency[4] Associated with levels of vitamin D[3] | NADSYN1^[[9]](#footnote-9)^ | rs7944926 rs12791871 rs2276362 rs3750997 rs2282621 rs3794060 rs12797951 rs12800438 rs1790345 | rs7944926  rs12791871 | chr11:70843273  chr11:70842192 |
| **rs3829251** | Associated with levels of vitamin D[2, 3] | NADSYN1 | rs10898193 rs11234042 rs1790349  rs7940244 | rs3829251 | chr11:70872207 |
| **rs2302190** | Associated with vitamin D level (not found in article text)[5] | MTMR4^[[10]](#footnote-10)^ | rs7207286 rs11650710 rs3744111 rs9903050 rs7209650 | rs2302190 | chr17:53939507 |
| **rs6730714** |  |  | None | None |  |
| **rs10508196** |  |  | None | None |  |
| **rs10507577** | Associated with vitamin D level[6] |  | rs2813582 | Omitted following quality control |  |

1. Moy KA, Mondul AM, Zhang H, Weinstein SJ, Wheeler W, Chung CC, Mannisto S, Yu K, Chanock SJ, Albanes D: **Genome-wide association study of circulating vitamin D-binding protein**. *The American journal of clinical nutrition* 2014, **99**(6):1424-1431.

2. Ahn J, Yu K, Stolzenberg-Solomon R, Simon KC, McCullough ML, Gallicchio L, Jacobs EJ, Ascherio A, Helzlsouer K, Jacobs KB *et al*: **Genome-wide association study of circulating vitamin D levels**. *Human molecular genetics* 2010, **19**(13):2739-2745.

3. Jorde R, Schirmer H, Wilsgaard T, Joakimsen RM, Mathiesen EB, Njolstad I, Lochen ML, Figenschau Y, Berg JP, Svartberg J *et al*: **Polymorphisms related to the serum 25-hydroxyvitamin D level and risk of myocardial infarction, diabetes, cancer and mortality. The Tromso Study**. *PloS one* 2012, **7**(5):e37295.

4. Wang TJ, Zhang F, Richards JB, Kestenbaum B, van Meurs JB, Berry D, Kiel DP, Streeten EA, Ohlsson C, Koller DL *et al*: **Common genetic determinants of vitamin D insufficiency: a genome-wide association study**. *Lancet* 2010, **376**(9736):180-188.

5. Anderson D, Holt BJ, Pennell CE, Holt PG, Hart PH, Blackwell JM: **Genome-wide association study of vitamin D levels in children: replication in the Western Australian Pregnancy Cohort (Raine) study**. *Genes Immun* 2014, **15**(8):578-583.

6. Benjamin EJ, Dupuis J, Larson MG, Lunetta KL, Booth SL, Govindaraju DR, Kathiresan S, Keaney JF, Jr., Keyes MJ, Lin JP *et al*: **Genome-wide association with select biomarker traits in the Framingham Heart Study**. *BMC Med Genet* 2007, **8 Suppl 1**:S11.

1. DBP: Vitamin-D-binding protein. [↑](#footnote-ref-1)
2. ST6GALNAC3: Gene encoding a sialyltransferase which might affect DBP synthesis, concentrations and function. [↑](#footnote-ref-2)
3. GC: Group specific component; gene encoding DBP. [↑](#footnote-ref-3)
4. EIA: Enzyme immunoassay. [↑](#footnote-ref-4)
5. NPY: Neuropeptide Y, neurotransmitter which mediates physiological processes including food intake and bone homeostasis. [↑](#footnote-ref-5)
6. According to UCSC Genome Browser on Human Dec. 2013 (GRCh38/hg38) assembly rs4751058 is located 391kb 5’ MGMT. MGMT encodes a DNA repair protein. [↑](#footnote-ref-6)
7. PDE3B: Phosphodiesterase 3B, together with vitamin D in a common pathway controlling lipid metabolism. [↑](#footnote-ref-7)
8. CYP2R1: Cytochrome P450, family2, subfamily R, polypeptide 1: Enzyme that converts vitamin D to 25(OH)D. [↑](#footnote-ref-8)
9. NADSYN1: Gene encoding Glutamine-dependent NAD(+) synthetase, which catalyzes the final step in the biosynthesis of NAD from nicotinic acid adenine dinucleotide (NaAD). Unknown effect on vitamin D. [↑](#footnote-ref-9)
10. MTMR4: Myotubalarin-related protein4: Dephosphorylates proteins phosphorylated on Ser, Thr, and Tyr residues and low molecular weight phosphatase substrate para-nitrophenylphosphate. Phosphorylates phosphatidylinositol 3,4,5-trisphosphate (PIP3). Unknown effect on vitamin D. [↑](#footnote-ref-10)
