# Supplementary material for: SNPs related to vitamin D and breast cancer risk: a case-control study
Source: Breast Cancer Res. 2018 Jan 2;20:1. doi: 10.1186/s13058-017-0925-3 (PMC5748964; doi:10.1186/s13058-017-0925-3)
Supplement: Supplementary file 2 — Baseline characteristics of control subjects and subjects with and without data on SNPs/vitamin D. (DOCX 24 kb) [file 13058_2017_925_MOESM2_ESM.docx]

Additional file 2:

| **Table S1: Baseline characteristics of controls and subjects with and without data on SNPs/vitamin D** | | | | | | | | | |  |
| --- | --- | --- | --- | --- | --- | --- | --- | --- | --- | --- |
| Factor | Category | Controls | |  | SNP-data | |  | Vitamin D-data | | |
|  |  | Cardiovascular cohort  (n=2658) | Matched controls  (n=704) |  | Available  (n=4058) | Not available  (n=250) |  | Available  (n=1343) | Not available  (n=2715) | |
|  |  | Column% | |  | Column% | |  | Column% | | |
| Age (years) | *Mean±standard deviation* | *57.49±5,95* | *57.06±7.26* |  | *57.22±6.51* | *57.32±6.31* |  | *56.92±7.26* | *57.37±6.10* | |
| Season of inclusion MDCS | Jan-March | 34.0 | 29.0 |  | 32.2 | 28.0 |  | 29.9 | 33.3 | |
|  | April-June | 17.6 | 24.4 |  | 20.2 | 23.6 |  | 23.9 | 18.4 | |
|  | July-Sept | 17.3 | 21.3 |  | 19.0 | 21.2 |  | 21.6 | 17.8 | |
|  | Oct-Dec | 31.1 | 25.3 |  | 28.6 | 27.2 |  | 24.6 | 30.6 | |
| Education | O-level college (7-9 ys.) | 75.5 | 68.2 |  | 72.1 | 75.6 |  | 67.8 | 74.2 | |
|  | A-level college (11-12 ys.) | 6.6 | 7.5 |  | 6.9 | 6.0 |  | 7.3 | 6.7 | |
|  | University | 17.7 | 24.3 |  | 20.8 | 18.4 |  | 24.7 | 18.9 | |
| Type of occupation | Manual worker | 40.2 | 37.9 |  | 38.1 | 35.6 |  | 36.5 | 39.0 | |
|  | Non-manual worker | 52.0 | 53.8 |  | 54.2 | 56.8 |  | 56.3 | 53.2 | |
|  | Employer/self-employed | 6.6 | 8.1 |  | 6.7 | 5.6 |  | 6.6 | 6.7 | |
|  | Missing | 1.2 | 0.1 |  | 1.0 | 2.0 |  | 0.7 | 1.1 | |
| Married/cohabitating | No | 31.0 | 32.7 |  | 31.6 | 35.6 |  | 33.1 | 30.8 | |
|  | Yes | 69.0 | 67.3 |  | 68.4 | 64.4 |  | 66.9 | 69.2 | |
| Age at menarche (years) | <12 | 6.6 | 4.4 |  | 6.5 | 4.8 |  | 6.0 | 6.8 | |
|  | 12-15 | 66.8 | 68.6 |  | 67.2 | 66.8 |  | 68.0 | 66.9 | |
|  | >15 | 25.6 | 26.4 |  | 25.3 | 28.0 |  | 25.2 | 25.3 | |
|  | Missing | 1.0 | 0.6 |  | 1.0 | 0.4 |  | 0.9 | 1.0 | |
| Parity | Nulliparous | 12.5 | 11.4 |  | 12.5 | 14.0 |  | 12.4 | 12.5 | |
|  | 1 child | 21.0 | 21.9 |  | 21.0 | 16.8 |  | 20.8 | 21.1 | |
|  | 2 children | 40.4 | 41.5 |  | 41.5 | 41.6 |  | 42.6 | 41.0 | |
|  | 3 children or more | 23.0 | 22.6 |  | 22.1 | 24.8 |  | 21.6 | 22.4 | |
|  | Missing | 3.1 | 2.7 |  | 2.9 | 2.8 |  | 2.6 | 3.0 | |
| Age at first birth (years) | Nulliparous | 12.5 | 11.4 |  | 12.5 | 14.0 |  | 12.4 | 12.5 | |
|  | ≤20 | 16.4 | 18.2 |  | 16.2 | 21.2 |  | 15.6 | 16.4 | |
|  | 21-24 | 28.0 | 27.1 |  | 28.0 | 24.0 |  | 27.7 | 28.1 | |
|  | 25-29 | 29.3 | 28.7 |  | 29.0 | 26.4 |  | 29.4 | 28.8 | |
|  | ≥30 | 10.7 | 11.9 |  | 11.5 | 11.6 |  | 12.2 | 11.1 | |
|  | Missing | 3.2 | 2.7 |  | 2.9 | 2.8 |  | 2.6 | 3.1 | |
| Separate missing categories given only if missing ≥1% | | | | | | | | | | |

| **Table S1: *continued*** | | | | | | | | | |
| --- | --- | --- | --- | --- | --- | --- | --- | --- | --- |
| Factor | Category | Controls | |  | SNP-data | |  | Vitamin D-data | |
|  |  | Cardiovascular cohort  (n=2658) | Matched controls  (n=704) |  | Available  (n=4058) | Not available  (n=250) |  | Available  (n=1343) | Not available  (n=2715) |
|  |  | Column% | |  | Column% | |  | Column% | |
| Age at menopause (years) | Pre-/Perimenopause | 25.6 | 32.1 |  | 29.0 | 28.8 |  | 32.8 | 27.1 |
|  | <45 | 10.4 | 8.9 |  | 9.6 | 13.6 |  | 9.0 | 9.9 |
|  | 45-53 | 46.0 | 42.2 |  | 44.1 | 39.2 |  | 42.1 | 45.1 |
|  | >53 | 16.3 | 15.5 |  | 15.5 | 18.0 |  | 14.3 | 16.1 |
|  | Missing | 1.7 | 1.3 |  | 1.7 | 0.4 |  | 1.8 | 1.7 |
| Exposure to oral contraceptives | No | 55.5 | 51.1 |  | 52.5 | 56.8 |  | 49.3 | 54.1 |
|  | Yes | 44.5 | 48.9 |  | 47.4 | 43.2 |  | 50.6 | 45.9 |
| Exposure to hormonal replacement therapy (HRT) | No (premenopausal) | 20.8 | 26.0 |  | 23.4 | 23.2 |  | 26.0 | 22.1 |
|  | No (postmenopausal) | 61.9 | 54.0 |  | 56.6 | 58.0 |  | 49.4 | 60.1 |
|  | Oestrogen only | 7.1 | 8.8 |  | 7.6 | 6.4 |  | 8.3 | 7.2 |
|  | Progesterone only | 0.5 | 0.9 |  | 0.7 | 0.8 |  | 1.1 | 0.5 |
|  | Combined HRT | 9.3 | 9.9 |  | 11.3 | 11.2 |  | 14.8 | 9.6 |
| Height (m) | ≤1.59 | 25.3 | 23.3 |  | 23.8 | 26.0 |  | 21.7 | 24.8 |
|  | 1.60-1.69 | 57.7 | 59.1 |  | 58.8 | 57.6 |  | 60.4 | 58.0 |
|  | ≥1.70 | 17.0 | 17.6 |  | 17.4 | 16.4 |  | 17.9 | 17.1 |
| Body mass index (kg/m^2^) | <25 | 53.8 | 51.7 |  | 52.4 | 59.6 |  | 50.9 | 53.1 |
|  | ≥25<30 | 33.0 | 36.2 |  | 34.1 | 30.8 |  | 34.9 | 33.6 |
|  | ≥30 | 13.1 | 12.1 |  | 13.5 | 9.6 |  | 14.2 | 13.2 |
| Alcohol consumption | Nothing last year | 11.4 | 11.2 |  | 11.0 | 10.0 |  | 10.9 | 11.1 |
|  | Something last year | 12.9 | 12.5 |  | 12.4 | 11.6 |  | 11.9 | 12.7 |
|  | Something last month | 75.4 | 76.0 |  | 76.3 | 78.4 |  | 77.0 | 75.9 |
| Smoking | Never | 47.6 | 42.3 |  | 46.0 | 38.8 |  | 43.3 | 47.4 |
|  | Current | 26.2 | 27.8 |  | 26.7 | 28.0 |  | 27.2 | 26.5 |
|  | Ex | 26.1 | 29.8 |  | 27.2 | 33.2 |  | 29.6 | 26.0 |
| Separate missing categories given only if missing ≥1% | | | | | | | | | |
